# GRP78 and Integrins Play Different Roles in Host Cell Invasion during Mucormycosis

**DOI:** 10.1128/mBio.01087-20

**Published:** 2020-06-02

**Authors:** Abdullah Alqarihi, Teclegiorgis Gebremariam, Yiyou Gu, Marc Swidergall, Sondus Alkhazraji, Sameh S. M. Soliman, Vincent M. Bruno, John E. Edwards, Scott G. Filler, Priya Uppuluri, Ashraf S. Ibrahim

**Affiliations:** a Division of Infectious Diseases, The Lundquist Institute for Biomedical Innovation, Harbor-University of California Los Angeles Medical Center, Torrance, California, USA; b David Geffen School of Medicine, University of California Los Angeles, Los Angeles, California, USA; c Research Institute for Medical and Health Sciences, College of Pharmacy, University of Sharjah, Sharjah, United Arab Emirates; d Institute for Genome Sciences, University of Maryland School of Medicine, Baltimore, Maryland, USA; Duke University Medical Center

**Keywords:** GRP78, integrin β1, *Rhizopus*, mucormycosis, cell invasion, epithelial cells, mice

## Abstract

Mucormycosis, caused by *Rhizopus* species, is a life-threatening fungal infection that occurs in patients immunocompromised by diabetic ketoacidosis (DKA), cytotoxic chemotherapy, immunosuppressive therapy, hematologic malignancies, or severe trauma. Inhaled *Rhizopus* spores cause pulmonary infections in patients with hematologic malignancies, while patients with DKA are much more prone to rhinoorbital/cerebral mucormycosis. Here, we show that *Rhizopus delemar* interacts with glucose-regulated protein 78 (GRP78) on nasal epithelial cells via its spore coat protein CotH3 to invade and damage the nasal epithelial cells. Expression of the two proteins is significantly enhanced by high glucose, iron, and ketone body levels (hallmark features of DKA), potentially leading to frequently lethal rhinoorbital/cerebral mucormycosis. In contrast, *R. delemar* CotH7 recognizes integrin β1 as a receptor on alveolar epithelial cells, causing the activation of epidermal growth factor receptor (EGFR) and leading to host cell invasion. Anti-integrin β1 antibodies inhibit *R. delemar* invasion of alveolar epithelial cells and protect mice from pulmonary mucormycosis. Our results show that *R. delemar* interacts with different mammalian receptors depending on the host cell type. Susceptibility of patients with DKA primarily to rhinoorbital/cerebral disease can be explained by host factors typically present in DKA and known to upregulate CotH3 and nasal GRP78, thereby trapping the fungal cells within the rhinoorbital milieu, leading to subsequent invasion and damage. Our studies highlight that mucormycosis pathogenesis can potentially be overcome by the development of novel customized therapies targeting niche-specific host receptors or their respective fungal ligands.

## Abstract (precis)

Mucormycosis caused by *Rhizopus* species is a fungal infection with often fatal prognosis. Inhalation of spores is the major route of entry, with nasal and alveolar epithelial cells among the first cells that encounter the fungi. In patients with hematologic malignancies or those undergoing cytotoxic chemotherapy, *Rhizopus* causes pulmonary infections. On the other hand, DKA patients predominantly suffer from rhinoorbital/cerebral mucormycosis. The reason for such disparity in disease types by the same fungus is not known. Here, we show that the unique susceptibility of DKA subjects to rhinoorbital/cerebral mucormycosis is likely due to specific interaction between nasal epithelial cell GRP78 and fungal CotH3, the expression of which increases in the presence of host factors present in DKA. In contrast, pulmonary mucormycosis is initiated via interaction of inhaled spores expressing CotH7 with integrin β1 receptor, which activates EGFR to induce fungal invasion of host cells. These results introduce a plausible explanation for disparate disease manifestations in DKA versus those in hematologic malignancy patients and provide a foundation for development of therapeutic interventions against these lethal forms of mucormycosis.

## INTRODUCTION

Mucormycosis is a lethal infection caused by mold belonging to the order Mucorales (1, 2). The infection is characterized by high degree of angioinvasion, which results in substantial tissue necrosis, frequently mandating surgical debridement of infected tissues (3, 4). Despite aggressive treatment with surgical removal of infected foci and use of the limited options of antifungal agents, mucormycosis is associated with dismal mortality rates of 50% to 100% (5, 6). Also, surviving patients often require major reconstructive surgeries to manage the ensuing highly disfiguring defects (2, 7).

*Rhizopus* spp. are the most common etiologic agents of mucormycosis, responsible for approximately 70% of all cases (1, 2, 6). Other isolated organisms belong to the genera *Mucor* and *Rhizomucor*, while fungi such as *Cunninghamella*, *Lichthemia*, and *Apophysomyces* less commonly cause infection (6). These organisms are ubiquitous in nature, found on decomposing vegetation and soil, where they grow rapidly and release large numbers of spores that can become airborne. While spores are generally harmless to immunocompetent people, almost all human infections occur in the presence of some underlying immunocompromising condition. These include hematological malignancies, organ or bone marrow transplant, corticosteroid use, hyperglycemia, diabetic ketoacidosis (DKA), and other forms of acidosis (2, 4, 8). Immunocompetent individuals suffering from burn wounds or severe trauma (e.g., soldiers in combat operations and motorcycle accident victims), or those injured in the aftermath of natural disasters (e.g., the Southeast Asian tsunami in 2004, or the tornadoes in Joplin, MO, in June 2011), are also uniquely susceptible to life-threatening Mucorales infections (9–11).

Devastating rhinoorbital/cerebral and pulmonary mucormycosis are the most common manifestations of the infection caused by the inhalation of spores (8, 12). In healthy individuals, cilia carry spores to the pharynx, which are later cleared through the gastrointestinal tract (13). Diabetes is a risk factor that predominantly predisposes individuals to rhinoorbital/cerebral mucormycosis (RCM) (6, 8). In susceptible individuals, RCM usually begins in the paranasal sinuses, where the organisms adhere to and proliferate in the nasal epithelial cells. Eventually, adhered Mucorales invade adjoining areas such as the palate, the orbit, and the brain, causing extensive necrosis, destruction of nasal turbinates, cranial nerve palsies, and facial disfigurement, all in a short span of days to weeks. Due to the angioinvasive nature of the disease, the infection often hematogenously disseminates to infect distant organs. We have shown that *Rhizopus* thrives under high-glucose and acidic conditions and can invade human umbilical vein endothelial cells via interaction of the fungal ligand, spore coat protein (CotH), with the host cell receptor glucose regulated protein 78 kDa protein (GRP78) (14, 15). In contrast, in neutropenic patients, inhaled spores can directly progress into the bronchioles and alveoli, causing pneumonia, and rarely cause RCM (13, 16, 17). The reasons why patients with DKA are mainly infected with RCM whereas neutropenic patients commonly suffer from pulmonary infections (8, 18) are not understood. We postulate that Mucorales ligands recognize host receptors unique to individual cell types (i.e., alveolar, nasal, and endothelial cells) and that this fungal ligand-host receptor interaction is enhanced by host factors, eventually leading to infections in the respective host niches.

To investigate this hypothesis, we identified the nasal and alveolar epithelial cell receptors to Mucorales ligands and studied the effect of host factors commonly present in DKA patients on the expression and interaction of these receptors/ligands. Here we show that, similarly to that on endothelial cells, the fungal CotH3 protein physically interacts with GRP78 on nasal epithelial cells. Elevated concentrations of glucose, iron, and ketone bodies present during DKA significantly induce the expression of GRP78 and CotH3, leading to enhanced invasion and damage of nasal epithelial cells. Antibodies against either CotH3 or GRP78 abrogate Rhizopus delemar invasion and damage of nasal epithelial cells. In contrast, *Rhizopus* binds to integrin β1 during invasion of alveolar epithelial cells. Binding to integrin β1 triggers the activation of epidermal growth factor receptor (EGFR) signaling (19). Anti-integrin β1 antibodies significantly reduce EGFR activation, block alveolar epithelial cell invasion, and protect neutropenic mice from pulmonary mucormycosis. These results introduce a plausible explanation for the unique susceptibility of DKA patients to RCM in which inhaled Mucorales spores are trapped in the sinuses via GRP78/CotH3 overexpression. We also posit that receptors identified in this study are potential novel targets for the development of pharmacologic or immunotherapeutic approaches against a variety of extremely lethal mucormycosis infections.

## RESULTS

### Distinct host receptors are used by *R. delemar* to invade and damage nasal or alveolar epithelial cells.

**FIG 1:**
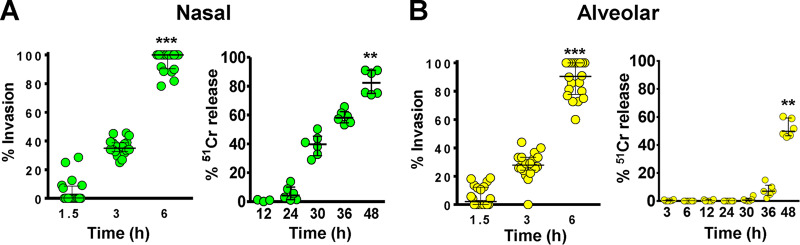
*R. delemar*-mediated invasion and damage of nasal and alveolar epithelial cells. *R. delemar* invasion of nasal (A) or alveolar (B) epithelial cells was determined using differential fluorescence assays by staining with 1% Uvitex for 1 h, while the damage assay was performed using the ^51^Cr release method. ***, *P* < 0.0001; **, *P* < 0.001 compared to the first time point in each panel. Data are presented as medians ± interquartile ranges from 3 independent experiments.

We compared the ability of *R. delemar* to invade and damage nasal and alveolar A549 epithelial cells *in vitro*. Incubation of *R. delemar* germlings with either of the two cell lines resulted in ∼40% invasion of host cells within the first 3 h of interaction, and by 6 h, almost all germlings had invaded the nasal and alveolar epithelial cells (Fig. 1). Interestingly, *R. delemar*-mediated damage of nasal epithelial cells occurred significantly earlier than damage of alveolar epithelial cells. Specifically, fungal germlings damaged 40% and 80% of the nasal epithelial cells within 30 h and 48 h, respectively (Fig. 1A). In contrast, no detectable damage and only 50% of the alveolar epithelial cells were injured after similar periods of incubation with *R. delemar* (Fig. 1B). These results also show that fungal invasion precedes damage of both types of epithelial cells. Importantly, *R. delemar-*mediated damage of primary human alveolar epithelial cells was similar to damage caused to A549 cells (see Fig. S1 in the supplemental material). Therefore, the invasion and damage of the alveolar epithelial cell line is reflective of *R. delemar* interactions with primary alveolar epithelial cells.

10.1128/mBio.01087-20.1 FIG S1 *R. delemar* damage of primary human alveolar epithelial cells (HPAEpiC). Damage assay time points were carried out using the ^51^Cr release method. Data are expressed as medians ± interquartile ranges. Download FIG S1, TIF file, 0.6 MB. Copyright © 2020 Alqarihi et al. 2020 Alqarihi et al. This content is distributed under the terms of the Creative Commons Attribution 4.0 International license.

**FIG 2:**
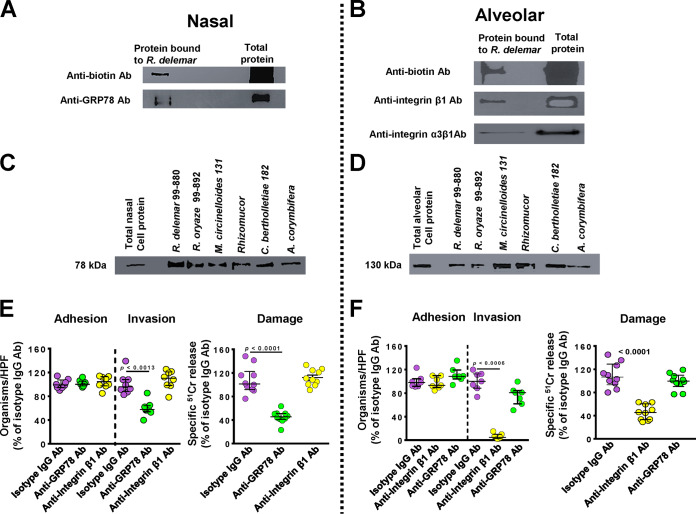
GRP78 is a nasal epithelial cell receptor, while integrin α3β1 is an alveolar epithelial cell receptor during Mucorales interaction. Biotinylated nasal (A) or alveolar (B) epithelial cells were incubated with *R. delemar* germlings, and unbound proteins were removed with repeated washing. Bound proteins were separated by SDS-PAGE and identified by Western blotting using an anti-biotin monoclonal antibody (Ab; top); the identity of the proteins was confirmed to be GRP78 (78 kDa) for nasal (A) or integrin β1 (130 kDa) (B) by using anti-GRP78 or anti-integrin α3β1 antibodies, respectively (bottom). Affinity purification of GRP78 (C) or integrin β1 (D) by other Mucorales. Anti-GRP78 and anti-integrin antibodies block *R. delemar*-mediated invasion and subsequent damage of nasal (E) and alveolar (F) epithelial cells, respectively, compared to that with the isotype matched-IgG. Both antibodies had no effect on adherence of the fungus to host cells. Data in panels E and F are expressed as medians ± interquartile ranges from 3 independent experiments. Different colors were used to simplify the graph: purple, isotype IgG; green, anti-GRP78 Ab; and yellow, anti-integrin β1 Ab.

We questioned if the disparity in damage to the two different epithelial cells was due to *R. delemar*’s ability to recognize different host receptors on the nasal and alveolar epithelial cells. We used an affinity purification process developed by Isberg and Leong (20), where *R. delemar* germlings were incubated separately with extracts of biotin-labeled total proteins of the nasal or alveolar epithelial cells. *R. delemar* specifically bound to a single nasal epithelial cell protein band that was isolated on an SDS-PAGE gel, and observed as a 78-kDa band after immunoblotting with anti-biotin antibodies (Fig. 2A). This protein band was identified by liquid chromatography-mass spectrometry (LC-MS) as the human GRP78, which we previously reported to be a receptor for invading Mucorales on human umbilical vein endothelial cells (14). To confirm the identity of the band, we stripped and probed the same immunoblot containing the nasal epithelial cell membrane proteins with anti-GRP78 polyclonal antibodies. The polyclonal antibodies recognized the 78-kDa band that had bound to *R. delemar* germlings (Fig. 2A).

Similarly, only a single 130-kDa protein band from the alveolar epithelial cell extracts was bound to *R. delemar* germlings (Fig. 2B). This protein was identified as integrin β1 by LC-MS and subsequently confirmed by probing with an anti-integrin β1 antibody on a Western blot (Fig. 2B). Integrins are known to be highly expressed in human lung tissues (https://www.ncbi.nlm.nih.gov/gene/3675), and we found that gene expression of integrin β1, but not GRP78, was upregulated in alveolar epithelial cells during infection with *R. delemar* (see Fig. S2). Furthermore, transcriptomic analysis of mouse lung tissues in early stages of pulmonary mucormycosis identified the upregulation of a gene encoding integrin α3 (21). Since integrins function as heterodimers (22), we sought to verify if integrin α3 subunit combines with integrin β1 in alveolar epithelial cells to act as a putative receptor for *R. delemar*. An integrin α3β1 polyclonal antibody recognized the 130-kDa band from A549 alveolar epithelial cells (Fig. 2B). Therefore, it is possible that the α3 subunit functions as a heterodimer with β1 to serve as a receptor during Mucorales invasion of alveolar epithelial cells.

10.1128/mBio.01087-20.2 FIG S2 Expression of GRP78 and integrin during *R. delemar* infection of alveolar epithelial cells after 3 h of interaction. The expression was quantified by qRT-PCR. Data are expressed as medians ± interquartile ranges from three independent experiments. Download FIG S2, TIF file, 0.6 MB. Copyright © 2020 Alqarihi et al. 2020 Alqarihi et al. This content is distributed under the terms of the Creative Commons Attribution 4.0 International license.

To investigate if nasal GRP78 and alveolar integrin α3β1 are putative universal receptors to other Mucorales, we performed the affinity purification experiment using germlings of other Mucorales clinical isolates. Indeed, all tested Mucorales, including Rhizopus oryzae 99-892, Mucor circinelloides 131, *Rhizomucor*, Cunninghamella bertholletiae 182, and Lichtheimia corymbifera 008-0490, bound GRP78 and integrin α3β1 from nasal (Fig. 2C) and alveolar (Fig. 2D) epithelial cells, respectively. Collectively, these data suggest that Mucorales interact with nasal and alveolar epithelial cells by using different host receptors.

### GRP78 and integrin β1 are receptors on nasal and alveolar epithelial cells, respectively.

To confirm the function of GRP78 and integrin β1 on nasal and alveolar epithelial cells as receptors for *R. delemar*, we examined the effect of anti-GRP78 and anti-integrin β1 antibodies on *R. delemar-*mediated host cell adhesion, invasion, and subsequent damage. While incubating nasal epithelial cells with anti-GRP78 polyclonal antibodies resulted in ∼50% inhibition of *R. delemar*-mediated host cell invasion, the antibodies had no effect on adhesion compared to that from isotype-matched control antibodies (Fig. 2E). The anti-GRP78 antibodies also reduced *R. delemar*’s ability to injure nasal epithelial cells by ∼60%. As expected, anti-integrin β1 antibodies had no effect on *R. delemar-*mediated adhesion to and invasion and damage of nasal epithelial cells (Fig. 2E). In contrast, compared to that with isotype-matched control antibodies, the use of anti-integrin β1 antibodies, and not anti-GRP78 antibodies, almost completely abolished the ability of *R. delemar* to invade alveolar epithelial cells (>95% reduction in invasion) (Fig. 2F). Similarly to anti-GRP78 and nasal epithelial cells, anti-integrin β1 antibodies had no effect on the adherence of the fungus to alveolar epithelial cells (median adherence of 98%, 93%, and 108% for isotype-matched IgG, anti-GRP78 IgG, and anti-integrin β1 IgG, respectively; *P* > 0.1). Finally, only anti-integrin β1 antibodies decreased the mold-mediated damage to alveolar epithelial cells by ∼60% (Fig. 2F). Overall, these results highlight that GRP78 and integrin β1 act as major and specific receptors to *R. delemar* during invasion and subsequent damage of nasal and alveolar epithelial cells, respectively.

**FIG 3:**
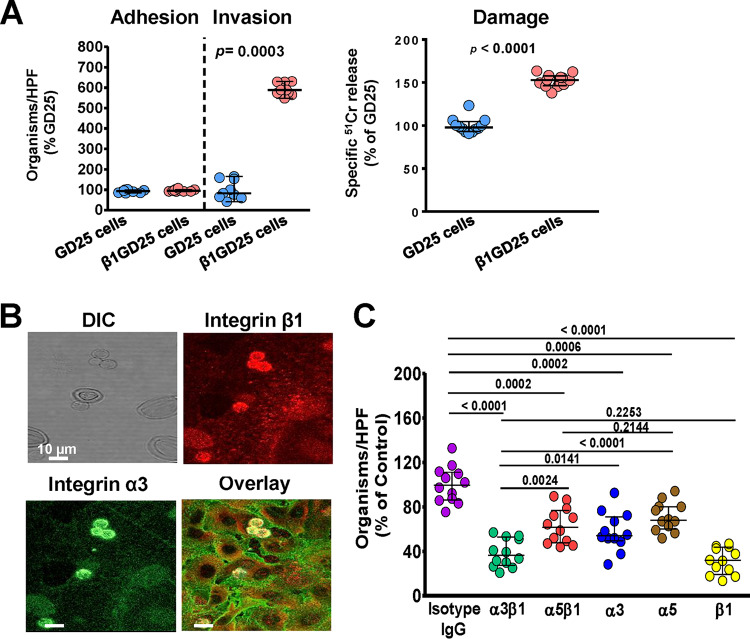
Integrin α3β1 is required for *R. delemar*-mediated host cell invasion and damage. (A) *R. delemar* has reduced invasion and damage of GD25 fibroblast cell line lacking integrin β1, compared to β1GD25, an integrin β1-restored fibroblast cell line. Adhesion and invasion of GD25 and β1GD25 fibroblast cell lines were assessed using differential fluorescence assays, while host cell damage was assessed using the ^51^Cr release method. (B) Confocal microscopy images showing the accumulation of integrin α3β1 around *R. delemar* during infection of alveolar epithelial cells. Images were taken after 2.5 h of incubation of the fungus with the host cells (C). Anti-integrin α3β1 monoclonal antibody blocks *R. delemar*-mediated invasion of alveolar epithelial cells. Alveolar epithelial cells were incubated with 5 μg/ml of different anti-integrin antibodies or isotype-matched IgG for 1 h prior to infection with *R. delemar.* Data in panels A and C are expressed as medians ± interquartile ranges from 3 independent experiments.

We previously demonstrated the importance of *R. delemar* interacting with GRP78 by overexpressing GRP78 on Chinese hamster ovarian cells (CHO) and showed increased *R. delemar-*mediated invasion and damage of the transfected cells (14). To validate the importance of integrin β1 as a receptor for *R. delemar* during invasion of alveolar epithelial cells, we compared the ability of *R. delemar* to invade and damage a GD25 fibroblast cell line generated from an integrin β1^−/−^ mouse by transfecting GD25 cells with mouse integrin β1 cDNA to produce the β1GD25 cell line (23). Despite the lack of difference in adhesion of *R. delemar* to these two cell lines, the β1GD25 fibroblast cells expressing integrin β1 were more susceptible to *R. delemar*-mediated invasion and damage than GD25 cells lacking integrin β1 (an increase of ∼600% for invasion and 150% for damage of β1GD25 versus that for GD25 cells) (Fig. 3A). These data reaffirm the importance of integrin β1 as a host receptor for *R. delemar* during invasion and subsequent damage of alveolar epithelial cells.

For cell membrane proteins to act as host cell receptors, they must be in close proximity to invading fungal cells. Therefore, we used an indirect immunofluorescence assay to localize integrin α3β1 on alveolar epithelial cells during infection with *R. delemar* germlings. Both integrin α3 (stained with an anti-integrin α3 antibody fluorescing green) and β1 (stained with an anti-integrin β1 antibody fluorescing red) were expressed on the surfaces of alveolar epithelial cells and coalesced on invading *R. delemar* germlings, with overlay images showing clear intense yellow staining around the fungal cells (Fig. 3B).

We previously showed that the filamentous fungal pathogen Aspergillus fumigatus invades alveolar epithelial cells through the fungus CalA protein binding to integrin α5β1 (24). Thus, to evaluate the function of integrin α5 as a potential receptor for *R. delemar*, we repeated the indirect immunofluorescence assay using antibodies targeting integrins β1 and α5. As expected, integrin β1 accumulated as a distinct ring-like formation around endocytosed *R. delemar* germlings. In contrast, integrin α5 had diffuse staining without accumulating around invading germlings (see Fig. S3). Thus, these data strongly suggest that the receptor for *R. delemar* during invasion of alveolar epithelial cells is likely to be integrin α3β1 rather than α5β1.

10.1128/mBio.01087-20.3 FIG S3 Colocalization of integrin β1 and α5 around *R. delemar*. Confocal microscopy images showing differentially fluorescent integrin β1 but not α5 around *R. delemar* germlings during infection of alveolar epithelial cells. Images were taken after 2.5 h of incubation of the fungus with the host cells. Download FIG S3, TIF file, 1.3 MB. Copyright © 2020 Alqarihi et al. 2020 Alqarihi et al. This content is distributed under the terms of the Creative Commons Attribution 4.0 International license.

To confirm the identity of the alveolar epithelial cell receptor during Mucorales invasion, we incubated the *R. delemar* germlings with A549 epithelial cells in the presence of specific monoclonal antibodies targeting either integrin α3, α5, or β1 separately and the two dimers of integrin α3 β1 or α5 β1. While all treatments resulted in a reduction of cellular invasion compared to that with the isotype-matched IgG antibodies (which did not block invasion), there were differences in the extent of invasion inhibition. Specifically, targeting integrin β1 caused the greatest reduction in invasion, with ∼70% inhibition, while anti-integrin α3 and anti-integrin α5 antibodies individually provided ∼50% and 30% protection from invasion, respectively (Fig. 3C). Interestingly, targeting both integrin α3 and β1 resulted in similar inhibition of *R. delemar* invasion of A549 cells as that provided by the anti-β1 antibody (∼70%) and significantly more than the invasion inhibition generated by the anti-α3 antibody or anti-α5β1 (Fig. 3C). Collectively, these results show that integrin β1 is the major host receptor acting as a heterodimer with α3 during *R. delemar* invasion of alveolar epithelial cells and that blocking these receptors can reduce *R. delemar* virulence to alveolar epithelial cells *in vitro*.

### Integrin β1 signaling is required for EGFR phosphorylation in alveolar epithelial cells during Mucorales infection.

**FIG 4:**
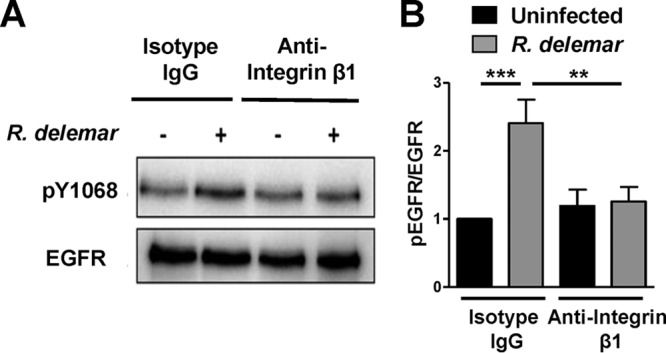
Anti-integrin antibodies block activation of alveolar epithelial cell EGFR. Representative immunoblots (A) and densitometric analysis (B) show that *R. delemar* infection induced phosphorylation of EGFR on tyrosine residue 1068 compared to that in the control and that anti-integrin β1 antibody blocked it. Data in panel B are means ± standard deviations from three independent experiments.

We recently reported that EGFR acts as a receptor for *R. delemar* during invasion of alveolar epithelial cells (19). However, the mechanism by which EGFR signaling is stimulated during infection was not identified. We tested if integrin β1 signaling played a role in stimulating EGFR activation during *R. delemar* invasion by examining phosphorylation of the A549 cells’ EGFR tyrosine residue 1068 in the presence of anti-integrin β1 antibodies. Using an immunoblotting assay, we determined that infection with *R. delemar* induces EGFR phosphorylation in A549 cells. When the *R. delemar*-A549 cell interaction was performed in the presence of integrin β1 antibodies, the phosphorylation of EGFR was abolished to basal levels (Fig. 4). Thus, these results are consistent with a model in which *R. delemar* interacts with integrin β1, causing activation of EGFR.

### *R. delemar* cell surface proteins CotH3 and CotH7 are the fungal ligands to nasal and alveolar epithelial cells, respectively.

**FIG 5:**
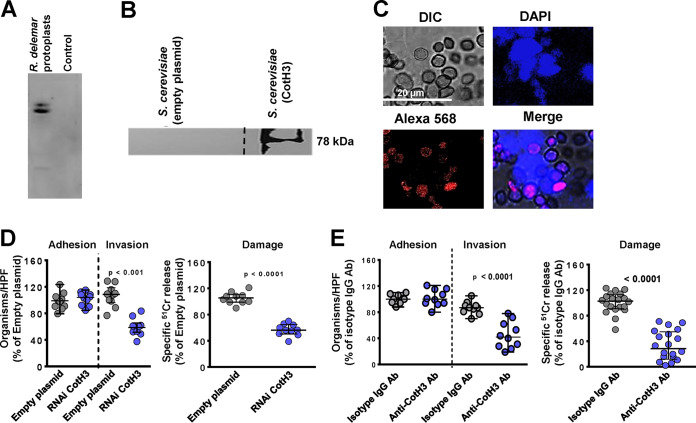
CotH3 is the *R. delemar* cell-surface ligand to GRP78 on nasal epithelial cells. (A) Far-Western blot of *R. delemar* surface proteins that bound to GRP78. (B) Affinity purification of nasal cell GRP78 by S. cerevisiae cells expressing CotH3 identified by anti-GRP78 antibody. Dashed line represents cropped image from GRP78 blot shown in Fig. 2A. (C) Confocal microscopy images showing interaction of nasal epithelial GRP78 and *R. delemar* CotH3 after a 2.5-h incubation shown by proximity ligation assay (PLA). DAPI staining was used to identify host cells. (D) Inhibition of CotH3 expression by RNAi reduced the ability of *R. delemar* to invade (by differential fluorescence) and damage (by ^51^Cr release method) nasal epithelial cells compared to that with empty plasmid-transformed *R. delemar*. Anti-CotH3 antibody blocked *R. delemar-*mediated invasion of and damage to nasal epithelial cells. Data in panels D and E are expressed as medians ± interquartile ranges from 3 independent experiments.

**FIG 6:**
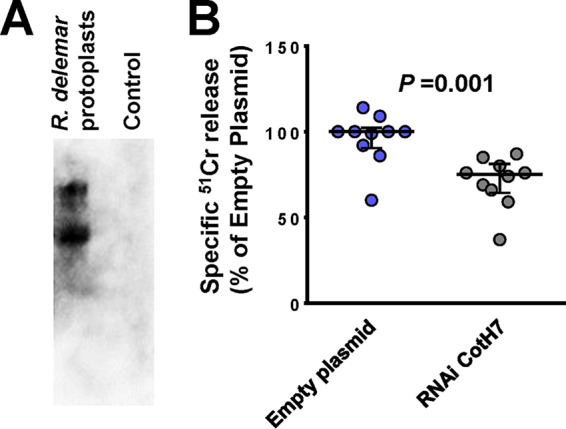
CotH7 is the *R. delemar* cell surface ligand to integrin α3β1. (A) Far-Western blot of *R. delemar* surface proteins that bound to integrin. (B) Inhibition of CotH7 expression by RNAi reduced the ability of *R. delemar* to damage alveolar epithelial cells compared to that with empty plasmid-transformed *R. delemar*. Data in panel B are expressed as medians ± interquartile ranges from 3 independent experiments.

Having identified the receptor on nasal and alveolar epithelial cells that interacts with *R. delemar* germlings, we next sought to identify the fungal cell surface protein that binds to GRP78 and integrin α3β1. Far-Western blot analysis using recombinant human GRP78 followed by anti-GRP78 antibodies or human integrin α3β1 followed by anti-integrin α3β1 antibodies identified the presence of prominent bands from the supernatant of *R. delemar-*regenerated protoplasts that bound to GRP78 (Fig. 5A) or integrin α3β1 (Fig. 6A). LC-MS of the bands identified CotH3 and CotH7 as putative fungal ligands binding to GRP78 and integrin α3β1, respectively. We previously described that CotH3 is the fungal ligand to host GRP78 during interaction of *R. delemar* with human umbilical vein endothelial cells (15, 25). Therefore, we used the tools available to us to determine the importance of CotH3 to *R. delemar* when interacting with nasal epithelial cells. We incubated biotinylated nasal epithelial cell membrane proteins with the model yeast Saccharomyces cerevisiae harboring a plasmid expressing CotH3 or S. cerevisiae expressing the empty plasmid as a negative control. The CotH3-expressing S. cerevisiae bound the 78-kDa protein of GRP78 as confirmed by Western blotting with anti-GRP78 antibodies, whereas the S. cerevisiae strain expressing empty plasmid did not (Fig. 5B). Next, we visualized the interaction between the two host fungal proteins by a proximity ligation assay (PLA). In this assay, nonfluorescent primary antibodies (commercially available) raised in different species are allowed to recognize GRP78 and CotH3 (using anti-CotH3 antibodies that we previously described [26]) on the host cells and fungus, respectively. Secondary antibodies directed against the constant regions of the two primary antibodies, called PLA probes, bind to the primary antibodies. The PLA probes fluoresce as a distinct bright spot only if the two proteins of GRP78 and CotH3 are in close proximity. Indeed, nasal epithelial cell-*R. delemar* germling interaction triggered the probe to fluoresce red (Fig. 5C). This fluorescence was located on germlings that interacted with host cells stained with 4′,6-diamidino-2-phenylindole (DAPI), yielding a bright pink color. Therefore, *R. delemar* CotH3 interacts with the GRP78 receptor on nasal epithelial cells, leading to invasion and subsequent damage of host cells.

To investigate if the interactions of CotH3 with GRP78 and CotH7 with integrin α3β1 mediate *R. delemar* invasion and damage of nasal and alveolar epithelial cells, we specifically downregulated the expression of CotH3 or CotH7 in *R. delemar* by RNA interference (RNAi). Individually targeting CotH3 and CotH7 by RNAi resulted in the generation of *R. delemar* mutants that had ∼90% (15) and 50% (see Fig. S4) inhibition in these two genes, respectively. Mutants were compared to the *R. delemar* strain transformed with an empty plasmid in their ability to invade and damage nasal and alveolar epithelial cells. Incubating nasal epithelial cells with an *R. delemar* RNAi-suppressed CotH3 strain displayed a >50% defect in invasion of and damage to nasal epithelial cells compared to that for the *R. delemar* strain transformed with the empty plasmid. The inhibition of CotH3 expression had no effect on the adherence of *R*. *delemar* to nasal epithelial cells (Fig. 5D) and did not affect the ability of *R. delemar* to interact with alveolar epithelial cells (see Fig. S5). Therefore, CotH3 is a specific *R. delemar* ligand that mediates invasion and subsequent damage to nasal epithelial cells.

10.1128/mBio.01087-20.4 FIG S4 RNAi targeting CotH7 inhibits the expression of CotH7. *R. delemar* was transformed with an RNAi construct targeting CotH7 expression or with an empty plasmid. Cells transformed with RNAi construct targeting CotH7 demonstrated 50% reduction in CotH7 expression relative to that in empty plasmid-transformed *R. delemar*, as determined by RT-PCR after 16 h of incubation. Download FIG S4, TIF file, 0.6 MB. Copyright © 2020 Alqarihi et al. 2020 Alqarihi et al. This content is distributed under the terms of the Creative Commons Attribution 4.0 International license.

10.1128/mBio.01087-20.5 FIG S5 Inhibition of CotH3 had no effect on invasion of or damage to alveolar epithelial cells by *R. delemar*. (A) Inhibition of CotH3 expression by RNAi did not alter the ability of *R. delemar* to adhere to, invade, or damage alveolar epithelial cells versus *R. delemar* transformed with empty plasmid. (B) Anti-CotH3 antibody blocked *R. delemar-*mediated adhesion and invasion but did not alter damage of alveolar cells compared to that of host cells incubated with isotype-matched IgG. Adhesion and invasion assays were carried out by differential fluorescence, while damage was assessed using the ^51^Cr release method. Data are expressed as medians ± interquartile ranges from 3 independent experiments. Download FIG S5, TIF file, 0.7 MB. Copyright © 2020 Alqarihi et al. 2020 Alqarihi et al. This content is distributed under the terms of the Creative Commons Attribution 4.0 International license.

We previously generated anti-CotH3 antibodies that blocked *R. delemar-*mediated invasion of endothelial cells. Therefore, we tested the ability of these antibodies to block *R. delemar-*mediated invasion of and subsequent damage to nasal epithelial cells. Anti-CotH3 antibodies resulted in 60% and 75% reduction in the ability of *R. delemar* to invade and damage nasal epithelial cells, respectively, compared to that with the isotype-matched control IgG (Fig. 5E). These results further confirm the importance of CotH3 protein in *R. delemar* interactions with nasal epithelial cells *in vitro.* Interestingly, anti-CotH3 antibodies reduced *R. delemar*-mediated invasion to alveolar cells compared to that with isotype-matched IgG (Fig. S5B).

Downregulation of CotH7 expression resulted in a statistically significant reduction (30% reduction) in *R. delemar-*mediated damage of alveolar epithelial cells (Fig. 6B). Similar to the outcome of the RNAi CotH3 mutant interacting with alveolar epithelial cells, downregulation of the CotH7 expression had no effect on *R. delemar* interacting with nasal epithelial cells (see Fig. S6). Therefore, interactions of *R. delemar* with alveolar epithelial cells are mainly driven by CotH7 binding to integrin α3β1.

10.1128/mBio.01087-20.6 FIG S6 Inhibition of CotH7 by RNAi had no effect on *R. delemar* interactions with nasal epithelial cells. Adhesion and invasion assays were conducted by differential fluorescence using nasal cells on 12-mm glass coverslips, while the damage assay was carried out using the ^51^Cr release assay. Data are expressed as medians ± interquartile ranges from 3 independent experiments. Download FIG S6, TIF file, 0.6 MB. Copyright © 2020 Alqarihi et al. 2020 Alqarihi et al. This content is distributed under the terms of the Creative Commons Attribution 4.0 International license.

### DKA host factors enhance *R. delemar-*mediate damage of nasal but not alveolar epithelial cells.

**FIG 7:**
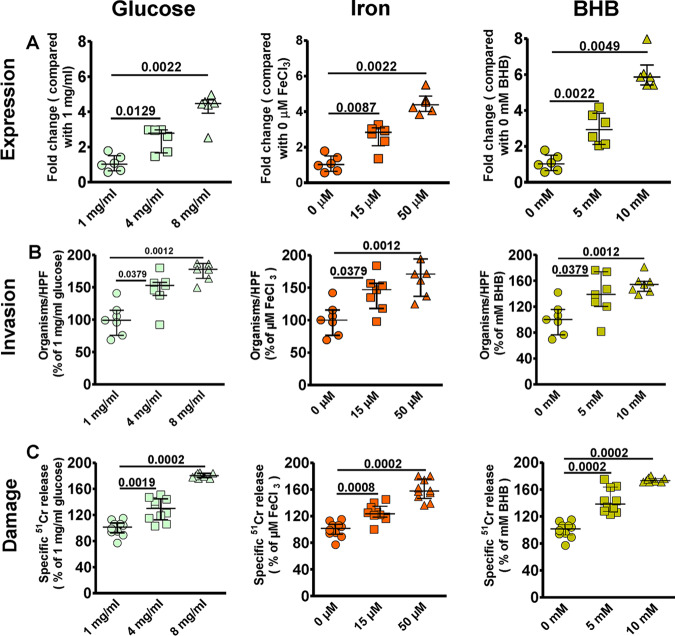
DKA host factors increase nasal epithelial cell GRP78 expression and host cell susceptibility to *R. delemar*-mediated invasion and damage. (A) Nasal epithelial cells were incubated with physiologically elevated concentrations of glucose, iron, or BHB for 5 h, and GRP78 gene expression was determined by quantitative reverse transcription-PCR (qRT-PCR). Elevated concentrations of glucose, iron, or BHB significantly enhanced *R. delemar*-mediated nasal epithelial cell invasion (B) and damage (C). Fold changes were calculated by comparison to the lowest concentration of the exogenous factors used. Data are expressed as medians ± interquartile ranges from 3 independent experiments.

**FIG 8:**
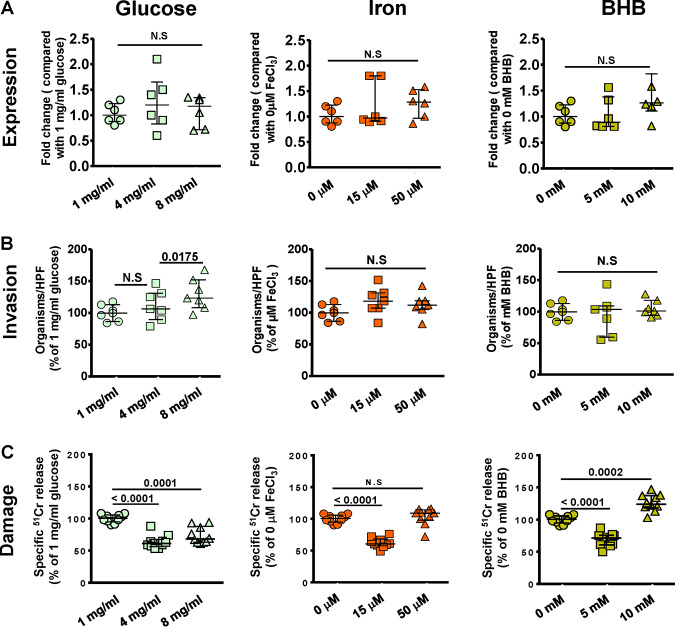
DKA host factors have no effect on integrin β1 expression levels and did not affect *R. delemar* interactions with alveolar epithelial cells. (A) Alveolar epithelial cells were incubated with physiologically elevated concentrations of glucose, iron, or BHB for 5 h, and integrin β1 gene expression was determined by qRT-PCR. Elevated concentrations of glucose, iron, or BHB had no effect on *R. delemar*-mediated alveolar epithelial cell invasion (B) or subsequent damage (C). Data are expressed as medians ± interquartile ranges from 3 independent experiments.

We previously showed that endothelial cell GRP78 and Mucorales CotH3 are overexpressed under the physiological conditions found in DKA patients such as hyperglycemia, elevated available serum iron, and high concentrations of ketone bodies, leading to enhanced invasion and damage of endothelial cells (14, 15, 25). Because we found that *R. delemar* uses a similar mechanism to interact with nasal epithelial cells, we reasoned that upregulation of GRP78 on nasal epithelial cells might lead to entrapment of inhaled spores in the nasal cavity of DKA patients, leading to rhinoorbital disease rather than pulmonary infection. To test this hypothesis, we measured the effect of physiologically elevated concentrations of glucose, iron, and β-hydroxy butyrate (BHB; as a representation for ketone bodies) on the GRP78 expression of nasal epithelial cells and subsequent interactions with *R. delemar.* The use of elevated concentrations of glucose (4 or 8 mg/ml), iron (15 to 50 μM of FeCl_3_), or BHB (5 to 10 mM) resulted in a ∼2- to 6-fold increase in the surface expression of GRP78 on nasal epithelial cells compared to that with normal concentrations of 1 mg/ml glucose, 0 μM iron, or 0 mM BHB (Fig. 7A). This enhanced expression of GRP78 coincided with increased ability of *R. delemar* to invade (Fig. 7B) and subsequently damage (Fig. 7C) nasal epithelial cells (∼150% to 170% increase in invasion and 120% to 170% in nasal epithelial cells damage versus that with the normal concentration of the effector). Conversely, the same elevated concentrations of glucose, iron, and BHB had no effect on the surface expression of integrin β1 of alveolar epithelial cells (Fig. 8A) and did not result in enhanced *R. delemar-*mediated invasion (with the exception of 8 mg/ml glucose, which caused a modest increase in invasion of 25% versus that with 1 mg/ml glucose) (Fig. 8B). Surprisingly, and in general, elevated concentrations of glucose, iron, or BHB resulted in 40% to 50% protection of alveolar epithelial cells from *R. delemar*-mediated injury (Fig. 8C). Collectively, these data suggest that nasal epithelial cells are more prone to *R. delemar-*mediated invasion and injury than alveolar epithelial cells when exposed to DKA host factors and likely explain, at least in part, the reason why DKA patients predominantly suffer from rhinoorbital rather than pulmonary mucormycosis.

### Anti-integrin β1 antibodies protect neutropenic mice from pulmonary mucormycosis.

**FIG 9:**
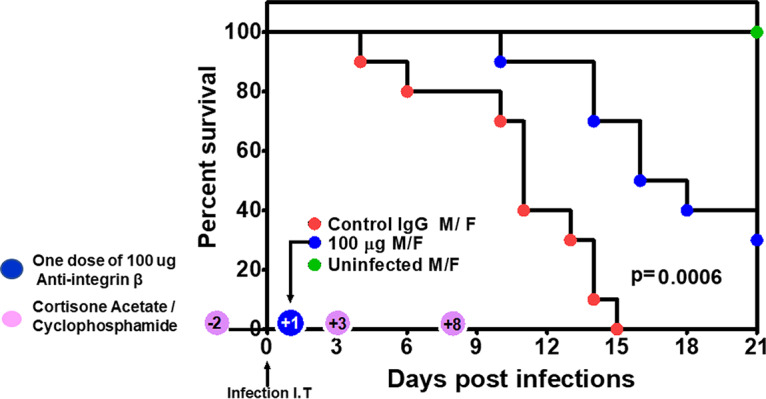
Anti-integrin β1 antibodies protect immunosuppressed mice from invasive pulmonary mucormycosis due to *R. delemar.* ICR mice (*n* = 10 [5 female and 5 male]/group with no difference in survival among the two sexes]) were immunosuppressed on days −2, +3, and +8 with cyclophosphamide and cortisone acetate and infected on day 0 intratracheally with *R. delemar* (actual inhaled inoculum of 2.8 × 10^3^/mouse). Twenty-four hours postinfection, mice were treated with a single dose of 100 μg of either an isotype-matched IgG (control) or an anti-integrin β1 antibody. *P* = 0.0006 by log rank test.

We previously showed that GRP78 can be targeted for treating experimental mucormycosis (14). To examine the potential of targeting integrins in treating pulmonary mucormycosis, we infected neutropenic mice intratracheally with *R. delemar* spores and treated them 1 day after infection with either an isotype-matched IgG or anti-integrin β1 polyclonal IgG. While mice treated with the isotype-matched IgG had a median survival time of 11 days and 100% mortality by day 15 postinfection, mice treated with the anti-integrin β1 IgG had an improved median survival time of 16 days, and 30% of the mice survived by day 21 postinfection when the experiment was terminated (Fig. 9). The surviving mice appeared healthy, and lungs and brains (primary and secondary target organs in this model [27]) harvested from the surviving mice had no residual infection as determined by lack of fungal growth from harvested organs when cultured on potato dextrose agar (PDA) plates. Thus, these data suggest that targeting integrin β1 should be explored to serve as a promising novel therapeutic option against pulmonary mucormycosis.

## DISCUSSION

**FIG 10:**
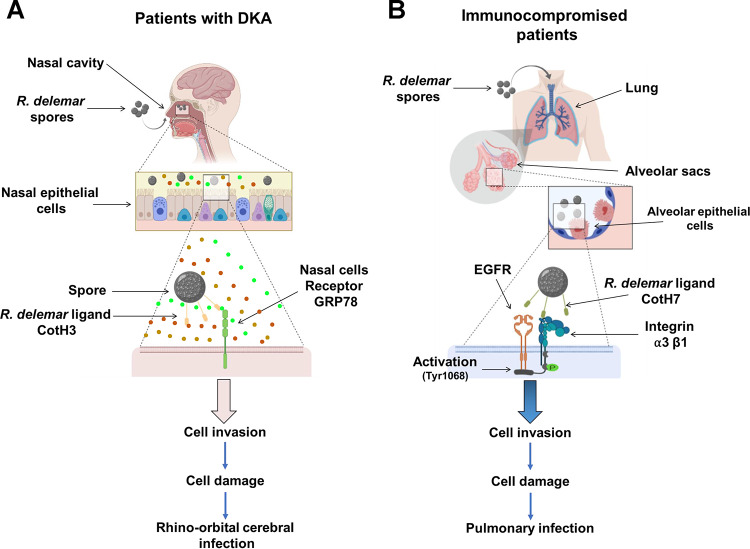
A diagram showing the molecular pathogenesis of the two main manifestations of mucormycosis. (A) *R. delemar* inhaled spores are trapped in the sinus cavities of patients with DKA due to the overexpression of GRP78 on nasal epithelial cells, and the interaction with fungal CotH3 results in rhinoorbital/cerebral mucormycosis. Colored circles represent elevated levels of glucose, iron, and ketone bodies. (B) In immunosuppressed patients, inhaled spores reach the alveoli and bind to integrin α3β1 via fungal CotH7, thereby triggering activation of EGFR and subsequent invasion and pulmonary infection.

Rhinoorbital/cerebral and pulmonary infections are the two most common manifestations of lethal mucormycosis (28). Despite acquiring the infection through inhaled spores, these two forms of disease manifestation are determined by the host’s underlying predisposing factors. Specifically, patients with DKA appear to be more likely than other susceptible hosts to have rhinoorbital/cerebral infection, while pulmonary mucormycosis afflicts neutropenic/leukemic hosts (2, 12, 29). Since the reason for this disparity is unknown (30), we questioned if Mucorales recognize host receptors expressed uniquely in distinct niches, especially in response to specific host environmental conditions. We previously found that the fungal cell surface CotH3 protein, a unique invasin to Mucorales fungi, binds to mammalian GRP78 when infecting and damaging umbilical vein endothelial cells (14, 15). Importantly, the expression of GRP78 host receptor and CotH3 fungal ligand increases several fold under physiological conditions present in the DKA patients, such as hyperglycemia, elevated iron, and ketoacidosis, leading to enhanced fungal invasion, subsequent damage of endothelial cells, and disease progression (25). Similar to these findings, we present multiple evidences by using affinity purification, specific antibody blocking, colocalization, and gene downregulation studies to show that *R. delemar* invades and damages nasal epithelial cells by CotH3 interacting with GRP78. As expected, DKA conditions of hyperglycemia, elevated iron, and ketoacidosis resulted in upregulation of GRP78 by nasal epithelial cells, causing enhanced fungal invasion. Therefore, in patients with DKA, inhaled Mucorales spores are likely trapped in the nasal milieu by the interaction of upregulated expression of GRP78-CotH3, resulting in rhinoorbital mucormycosis (Fig. 10A). The highly angioinvasive *R. delemar* can eventually spread from the damaged nasal epithelial cells into surrounding tissue vasculature by continuing to interact with GRP78 on endothelial cells (15, 25). In contrast, by using similar approaches, we show that the integrin α3β1 is the receptor for *R. delemar* on alveolar epithelial cells which activated EGFR, resulting in invasion and pulmonary infection (Fig. 10B). However, hyperglycemia, elevated iron, and ketoacidosis, as seen in DKA patients, did not increase integrin α3β1 expression on alveolar epithelial cells. In fact, through an unexplained mechanism(s), elevated physiological concentrations of glucose, iron, and BHB protected A549 cells from invasion and subsequent damage by *R. delemar*. The protection of alveolar epithelial cells from *R. delemar-*mediated invasion and subsequent damage when exposed to elevated glucose, iron, or BHB is likely to provide further explanation on why DKA patients rarely develop pulmonary disease. Future studies will investigate the mechanism by which DKA host factors protect alveolar epithelial cells from *R. delemar*-mediated invasion and damage.

One of the intriguing results is the difference of susceptibility of nasal and alveolar epithelial cells to *R. delemar*-mediated damage despite being equally susceptible to fungal invasion. Specifically, nasal epithelial cells were more susceptible to fungal damage than alveolar epithelial cells (Fig. 1). We previously reported on the role of *R. delemar* toxins in mediating damage to host cells (31). It is possible that the two cell types have distinct susceptibility and/or induce different levels and/or types of these toxins. Alternatively, binding to distinct receptors is likely to induce specific signal transduction pathways that might explain the differences in host cell death patterns. These possibilities are the topic of active investigation in our laboratory.

We previously reported on the CotH gene family which is uniquely and universally present in Mucorales fungi and required for mucormycosis pathogenesis (15, 21). Specifically, CotH3 mediates invasion of endothelial cells by binding to GRP78 (15, 25). *R. delemar* also uses CotH3 to invade nasal epithelial cells via binding to GRP78. However, in lung tissues where integrins are highly expressed (https://www.ncbi.nlm.nih.gov/gene/3675), CotH7 appears to be the major *R. delemar* ligand mediating binding to integrin α3β1 of alveolar epithelial cells. Although CotH2 and CotH3 proteins are closely related, CotH7 is distantly related, with ∼50% amino acid identity to CotH3 (see Fig. S7 in the supplemental material). It is noted that CotH2, CotH3, and CotH7 are among the most expressed genes in the entire genome of two clinical isolates (*R. delemar* 99-880 and *R. oryzae* 99-892), and their expression is not induced by alveolar epithelial cells (21). This noninduced high expression and the presence of altered protein family members is likely necessary for the organism to successfully infect host niches in which invasion of tissues is dictated by the presence of different receptors. However, in both nasal and alveolar epithelial cells, antibody blocking studies targeting the receptors or the ligands did not completely block *R. delemar-*mediated adhesion to and invasion or damage of host cells. Thus, other host receptors/fungal ligands are likely to be involved in these interactions.

10.1128/mBio.01087-20.7 FIG S7 The CotH protein family. Phylogenetic tree and relative distance of *R. delemar* CotH proteins (A) and their percent identity (B). Download FIG S7, TIF file, 0.8 MB. Copyright © 2020 Alqarihi et al. 2020 Alqarihi et al. This content is distributed under the terms of the Creative Commons Attribution 4.0 International license.

We found that anti-CotH3 antibodies, but not reduction of CotH3 expression by RNAi, were able to block invasion and, to a lesser extent, adherence of *R. delemar* to alveolar epithelial cells (Fig. S5). It is noted that the antibodies were generated against a peptide of CotH3 (MGQTNDGAYRDPTDNN [26]) that is ∼70% conserved in CotH7 protein (see Fig. S8), whereas the inhibition of CotH3 expression by RNAi resulted in ∼80% gene silencing (15).

10.1128/mBio.01087-20.8 FIG S8 Alignment results between CotH3 peptide (that has been used for anti-CotH3 production) and CotH7. Multiple Sequence Comparison by Log-Expectation (MUSCLE) online tool used to perform sequence alignment between 16-mer CotH3 and CotH7 proteins using the cluster 12.1 algorithm. Download FIG S8, TIF file, 0.7 MB. Copyright © 2020 Alqarihi et al. 2020 Alqarihi et al. This content is distributed under the terms of the Creative Commons Attribution 4.0 International license.

Integrins are a family of adhesion receptors consisting of α and β heterodimer transmembrane subunits that are specialized in binding cells to the extracellular matrix (22). They can also function as receptors for extracellular ligands and transduce bidirectional signals into and outside the cell using effector proteins (32, 33). One such pathway involves the ability of integrins to cooperate with EGFR, leading to synergy in cell proliferation, cell survival, and cell migration (34). We recently reported on the use of an unbiased survey of the host transcriptional response during early stages of *R. delemar* infection in a murine model of pulmonary mucormycosis as well as an *in vitro* A549 cell infection model by using transcriptome analysis sequencing. Transcriptome sequencing (RNA-seq) data showed an activation of the host’s EGFR by an unknown mechanism (19). Furthermore, an FDA-approved inhibitor of EGFR, gefitinib, successfully inhibited alveolar epithelial cell invasion by *R. delemar in vitro* and ameliorated experimental murine pulmonary mucormycosis (19). Our data highly suggest that activation of EGFR occurs by binding of the fungus to integrin β1 (Fig. 10B). Specifically, the use of an anti-integrin β1 antibody prevents the *R. delemar-*induced activation of EGFR.

We previously reported on protecting DKA mice from mucormycosis by using antibodies targeting GRP78-CotH3 interactions (14, 15, 26). In these studies, mice were partially protected when the antibodies were introduced alone, and maximal protection occurred when anti-CotH3 antibodies were combined with antifungal agents (26), indicating the potential translational benefit of this therapeutic approach. In this study, we also demonstrated partial but highly significant protection against pulmonary mucormycosis when a single administration of anti-integrin β1 was used. This antibody dose translates to ∼4.0 mg/kg of body weight, which is within the antibody doses currently in clinical practice of 1 to 15 mg/kg of body weight, thereby emphasizing the clinical applicability of this approach. One caveat of an immunotherapeutic approach targeting host cell receptors such as integrins or GRP78 is the potential host toxicity. However, it is prudent to point out that targets such as GRP78, integrins, or EGFR are the subject of developing and/or developed therapeutic strategies against cancer (34, 35). One advantage of developing therapies targeting integrins would be the possibility of using the developed therapy to treat aspergillosis, since we showed that integrin β1 was also identified as a host receptor on A549 alveolar epithelial cells when interacting with A. fumigatus cell surface protein CalA (24). A. fumigatus CalA specifically interacts with the integrin α5β1 subunit rather than integrin α3β1, the predominant receptor for *R. delemar*. However, blocking of integrin α5 or α5β1 also resulted in a modest yet detectable decrease in *Rhizopus* invasion of alveolar epithelial cells, indicating that the α5 subunit may play a minor role in fungal interactions. Therefore, a therapy that targets both infections would have to focus on targeting integrin β1. Finally, nasal and/or alveolar epithelial cell interactions are early steps of the disease, and any potential therapy targeting these interactions is likely to be more successful if initiated early on, preferably with antifungal therapy to block invasion and enhance fungal clearance. Unfortunately, diagnosis of mucormycosis often occurs in late-stage disease and is currently reliant on histopathology or nonspecific radiological methods (36). However, early results of several methods reliant on molecular diagnosis (including those targeting CotH genes [37–40]) and serology (targeting mannans [41]) are encouraging and likely to help in implementing early therapy.

To summarize, the unique susceptibility of DKA subjects to rhinocerebral mucormycosis is likely due to a specific interaction between nasal epithelial cell GRP78 and fungal CotH3, the expression of which increases in the presence of environmental factors present in DKA, which results in trapping inhaled spores in the nasal cavity. In contrast, pulmonary mucormycosis is initiated via interaction of inhaled spores expressing CotH7 with integrin α3β1 receptor, which activates EGFR to induce fungal invasion of host cells. These results add to our previously published line of evidence on the pathogenesis of mucormycosis in different hosts and provide the groundwork for the development of therapeutic interventions against lethal drug-resistant mucormycosis.

## MATERIALS AND METHODS

### *R. delemar* and culture conditions.

A variety of clinical Mucorales isolates were used in this study. *R. delemar* 99-880 (brain isolate from a patient with rhinocerebral mucormycosis), *R. oryzae* 99-892 (isolated from a patient with pulmonary mucormycosis), and Mucor circinelloides 131 were obtained from the fungus testing laboratories at University of Texas Health Science Center at San Antonio (UTHSCSA), Texas. Lichtheimia corymbifera strain 008-0490 and *Rhizomucor* were collected from patients enrolled in the Deferasirox-AmBisome Therapy for Mucormycosis (DEFEAT Mucormycosis) study (42). Cunninghamella bertholletiae 182 is a clinical isolate obtained from Thomas Walsh (Weill Cornell Medicine, NY, USA). Saccharomyces cerevisiae ATCC 62956 (LL-20) and its *his3*Δ and *leu*Δ mutants were constructed by L. Lau (University of Illinois at Chicago). S. cerevisiae expressing *R. delemar* CotH3 protein driven by the galactose-inducible promoter (15) was utilized to confirm the candidate ligand for the nasal epithelial cells. Mucorales were grown on PDA plates (BD Biosciences Diagnostic Systems) for 3 to 5 days at 37°C, while S. cerevisiae was grown on synthetic dextrose minimal medium (SD) for 3 to 5 days. All incubations were performed at 37°C. To induce the expression of CotH3 in S. cerevisiae, the yeast cells were grown in synthetic galactose minimal medium (SG) at 37°C for 16 h. The sporangiospores were collected in endotoxin-free Dulbecco’s phosphate-buffered saline (PBS) containing 0.01% Tween 80 for Mucorales, washed with PBS, and counted with a hemocytometer to prepare the final inoculum. For S. cerevisiae, cells were centrifuged, washed with PBS, and counted as described above.

To form germlings, spores were incubated in Kaighn’s modification of Ham’s F-12 medium (F-12K from the American Type Culture Collection [ATCC]) at 37°C with shaking for 1 to 3 h based on the assay under study. Germlings were washed twice with F-12 medium for all assays used, except in experiments involving isolation of the epithelial cell receptor, for which the germlings were washed twice with PBS (plus Ca^2+^ and Mg^2+^).

### Host cells.

Nasal epithelial cells (CCL-30) were obtained from ATCC and cultured in Eagle’s minimum essential medium (EMEM) supplemented with 10% fetal bovine serum and penicillin-streptomycin. Homo sapiens alveolar epithelial cells (A549 cells) procured from ATCC were obtained from a 58-year-old male Caucasian patient with carcinoma. They were propagated in F-12 medium developed for alveolar A549 epithelial cells. The GD25 and β1GD25 cell lines were obtained from Deane F. Mosher, University of Wisconsin—Madison. The cells were cultured to confluence in Falcon tissue culture treated flasks (75 cm^2^) at 37°C with 5% CO2. Primary alveolar epithelial cells were obtained from ScienCell (HPAEpiC; catalog no. 3200), propagated in alveolar epithelial cell medium (catalog no. 3201), and passaged once.

### Invasion of *R. delemar* to epithelial cells.

The number of organisms invading epithelial cells was determined using a modification of our previously described differential fluorescence assay (43). Briefly, 12-mm glass coverslips in 24-well cell culture plates were coated with fibronectin for at least 4 h and seeded with epithelial cells until confluence. After washing twice with prewarmed Hanks’ balanced salt solution (HBSS; Irvine Scientific), the cells were then infected with 2.5 × 10^5^ cells of *R. delemar* in F-12K medium that had been germinated for 2 h. Following incubation for 3 h, the cells were fixed in 3% paraformaldehyde and stained for 1 h with 1% Uvitex (Polysciences), which specifically binds to the chitin of the fungal cell wall. After washing 5 times with PBS, the coverslips were mounted on a glass slide with a drop of ProLong Gold antifade reagent and sealed with nail polish. The total number of cell-associated organisms (i.e., germlings adhering to monolayer) was determined by phase-contrast microscopy. The same field was examined by epifluorescence microscopy, and the number of uninternalized germlings (which were brightly fluorescent) was determined. The number of endocytosed organisms was calculated by subtracting the number of fluorescent organisms from the total number of visible organisms. At least 100 organisms were counted in 20 different fields on each slide. Two slides per arm were used for each experiment, and the experiment was performed in triplicates on different days.

### *R. delemar*-induced epithelial cell damage.

Host cell damage was quantified by using a chromium (^51^Cr)-release assay (44). Briefly, epithelial cells grown in 24-well tissue culture plates were incubated with 1 μCi per well of Na*_2_*^51^CrO_4_ (ICN) in EMEM or F12-K medium (for nasal or alveolar cells) for 16 h. On the day of the experiment, the unincorporated ^51^Cr was aspirated, and the wells were washed twice with warmed HBSS. Cells were infected with 2.5 × 10^5^ spores suspended in 1 ml in EMEM or F-12K medium. Spontaneous ^51^Cr release was determined by incubating epithelial cells in EMEM or F-12K medium without *R. delemar.* After 30 h of incubation of spores with nasal cells, or 48 h for alveolar cells, 50% of the medium was aspirated from each well and transferred to glass tubes. Approximately 500 μl of 6 N NaOH was added to each well and incubated for 15 min, and the medium was transferred from the wells to a glass tube. Subsequently, each well was rinsed with 500 μl of Radiacwash (Biodex), which was transferred to the same tube. The amount of ^51^Cr in the tubes was determined by gamma counting. The total amount of ^51^Cr incorporated by epithelial cells in each well equaled the sum of radioactive counts per minute of the aspirated medium plus the radioactive counts of the corresponding cells. After the data were corrected for variations in the amount of tracer incorporated in each well, the percentage of specific epithelial cell release of ^51^Cr was calculated by the following formula: [(experimental release) − (spontaneous release)]/[total incorporation − (spontaneous release)]. Each experimental condition was tested at least in triplicates, and the experiment was repeated at least once.

For antibody (Ab)-mediated blocking of adherence, invasion, or damage caused by *R. delemar*, the assays were carried out as described above except that epithelial cells were incubated with the respective antibodies (50 μg/ml anti-GRP78 or 5 μg/ml anti-integrin β1 or integrin α3β1 Ab or anti-IgG [as an isotype matching control]) for 1 h prior to addition of *R. delemar* germlings.

### Effect of acidosis, iron, glucose, or β-hydroxy butyrate on *R. delemar*-epithelial cell interactions.

Studies were performed to investigate the effect of glucose, iron, or BHB on epithelial cell GRP78 or integrin expression levels, and to test their impact on subsequent interactions of epithelial cells with *R. delemar* germlings. Epithelial cells were grown in EMEM or F-12K medium containing various concentrations of FeCl_3_, glucose, or BHB for 5 h. Invasion and damage assays were conducted as mentioned in the previous section. Quantitative RT-PCR was used to measure the expression of nasal GRP78 and alveolar integrin expression using the following primers: for GRP78, forward primer GGAAAGAAGGTTACCCATGC and reverse primer AGAAGAGACACATCGAAGGT; for integrin, forward primer GAAGGGTTGCCCTCCAGA and reverse primer GCTTGAGCTTCTCTGCTGTT. The gene expression data were normalized to the GAPDH housekeeping gene expression (forward primer ACCATCTTCCAGGAGCGAC and reverse primer TAAGCAGTTGGTGGTGCAG).

### Extraction of epithelial cell membrane proteins.

Epithelial cell membrane proteins were extracted according to the method of Isberg and Leong (20). Briefly, epithelial cells grown to confluence in 20 flasks of 75 cm^2^, were split into ten tissue culture dishes 150 mm by 25 mm, and incubated at 37°C in 5% CO_2_ until they reached confluence (typically 5 to 7 days). The cells were washed two times with 12 ml warm PBS containing Ca^2+^ and Mg^2+^ (PBS-CM) prior to incubating them with 0.5 mg/ml EZ-Link sulfo-NHS-LS-biotin (Pierce) (12 min in 5% CO_2_ at 37°C). Subsequently, the cells were then rinsed extensively with cold PBS-CM and scraped from the tissue culture dishes. The epithelial cells were collected by centrifugation at 500 × *g* for 5 min at 4°C and then lysed by incubation for 20 min on ice in PBS-CM containing 5.8% *n*-octyl-β-d-glucopyranoside (Fisher) and protease inhibitor cocktail solution (Fisher). The cell debris was removed by centrifugation at 5,000 × *g* for 5 min at 4°C. The supernatant was collected and centrifuged at 100,000 × *g* for 1 h at 4°C. The concentration of the epithelial cell proteins in the resulting supernatant was determined using the Bradford method (Bio-Rad).

### Isolation of epithelial cell receptors that bind to Mucorales.

Live Mucorales spores (8 × 10^8^) or an equivalent volume of 1- to 3-h germlings (approximately 1 × 10^8^ cells) were incubated for 1 h on ice with 250 μg of biotin-labeled epithelial cell surface proteins in PBS-CM plus 1.5% *n*-octyl-β-d-glucopyranoside and protease inhibitor cocktail. The unbound epithelial cell proteins were washed away by 5 rinses with this buffer. The epithelial cell proteins that remained bound to the fungal cells were eluted twice with 6 M urea (Sigma). The proteins were then separated by 10% SDS-PAGE and transferred to Immun-Blot polyvinylidene difluoride (PVDF) membrane (Bio-Rad). The membrane was then treated with Western blocking reagent (Roche) and probed with an anti-biotin horseradish peroxidase (HRP)-conjugated linked antibody (Cell Signaling). After incubation with SuperSignal West Dura extended duration substrate (Pierce), the signals were detected using a charge-coupled-device (CCD) camera.

To identify epithelial cell proteins that bound to Mucorales, we incubated epithelial cell membrane proteins with *R. delemar* germlings as described above. The eluted proteins were separated by SDS-PAGE, and the gel was stained with Instant Blue stain (Fisher). The major two bands at approximately 75 and 130 kDa (from nasal and alveolar cells, respectively) were excised and microsequenced using matrix-assisted laser desorption ionization–time of flight tandem mass spectrometry (MALDI-TOF MS/MS) (The Lundquist Institute Core Facility).

To confirm the identity of GRP78 and integrin α3β1, epithelial cell membrane proteins that bound to *R. delemar* were separated on an SDS-polyacrylamide gel and transferred to PVDF-plus membranes. Membranes were probed with a rabbit anti-GRP78 antibody (Abcam), followed by HRP-conjugated goat anti-rabbit IgG (Pierce) as a secondary antibody (for nasal cells) and rabbit anti-integrin α3β1 (Abcam), followed by HRP-conjugated goat anti-rabbit IgG (Pierce). After incubation with SuperSignal West Dura extended duration substrate (Pierce), the signals were detected using enhanced chemiluminescence and imaged with a C400 (Azure Biosystems) digital imager.

### Immunoblot of EGFR phosphorylation *in vitro*.

A549 cells in 24-well tissue culture plates were incubated in F-12K tissue culture medium supplemented with fetal bovine serum to a final concentration of 10%. Prior to infection, the A549 cells were serum starved for 120 min. Spores of *R. delemar* were incubated in RPMI medium for 60 min at 37°C, washed, and suspended in F-12K medium. A549 cells were infected for 3 h with a multiplicity of infection (MOI) of 5. Next, the cells were rinsed with cold HBSS containing protease and phosphatase inhibitors and removed from the plate with a cell scraper. After collecting the cells by centrifugation, they were boiled in 2× SDS sample buffer. The lysates were separated by SDS-PAGE, and Y1068 EGFR phosphorylation was detected with a phospho-specific antibody (Cell Signaling). The blots were then stripped, and total protein levels was detected by immunoblotting with appropriate antibodies against EGFR (Cell Signaling). The immunoblots were developed using enhanced chemiluminescence and imaged with a C400 (Azure Biosystems) digital imager.

### Colocalization of GRP78 and integrin α3β1 with phagocytosed *R. delemar* germlings.

We used a modification of our previously described method (14). Confluent epithelial cells on a 12-mm-diameter glass coverslip were infected with 2.5 × 10^5^ cells/ml *R. delemar* cells in EMEM or F12-K medium that had been pregerminated for 2 h. After 3 h of incubation at 37°C, the cells were gently washed twice with HBSS to remove unbound organisms and then fixed with 3% paraformaldehyde for 15 min.

For *R. delemar* interaction with nasal cells, a proximity ligation assay (PLA) technique (Sigma-Aldrich) was performed. For the PLA, two primary antibodies raised in different species are used to detect two unique protein targets. A pair of oligonucleotide-labeled secondary antibodies (PLA probes) then bind to the primary antibodies. Hybridizing connector oligonucleotides join the PLA probes only if they are in close proximity to each other, allowing for an up to 1,000-fold amplified signal tethered to the PLA probe, resulting in localization of the signal. This is visualized and quantified as discrete spots (PLA signals) by microscopy image analysis. Thus, two different antibodies were used: a mouse anti-GRP78 IgG was used to stain paraformaldehyde-fixed nasal cells, while anti-rabbit IgG against CotH3 was used to label *R. delemar*. Interactions between the two cell-surface proteins were carried out according to the kit instructions and visualized by confocal microscopy.

For alveolar epithelial cell-*R. delemar* interactions, the formaldehyde-fixed epithelial cell-spore mixture was incubated with 1% bovine serum albumin (BSA) for 1 h (blocking step). Next, cells were incubated with antibodies against integrin α3 or integrin β1 (eBioscience and Santa Cruz Biotechnology), followed by incubations with the appropriate secondary antibodies labeled with either Alexa Fluor 488 or Alexa Fluor 568 (Thermo Fisher Scientific). After washing, the coverslip was mounted on a glass slide with a drop of ProLong Gold antifade reagent (Molecular Probes and Invitrogen) and viewed by confocal microscopy. The final confocal images were produced by combining optical sections taken through the *z* axis.

### Protoplast formation and collection of *R. delemar* cell wall material.

To identify the *R. delemar* ligand that binds to epithelial cell GRP78, we collected cell wall material from supernatants of protoplasts of *R. delemar* germlings. Briefly, *R. delemar* spores (6 × 10^6^) were germinated in yeast extract-peptone-dextrose (YPD) medium for 3 h at 37°C. Germinated cells were collected by centrifugation at 900 × *g*, washed twice with 0.5 M sorbitol, and then resuspended in 0.5 M sorbitol in sodium phosphate buffer (pH 6.4). Protoplasting solution consisting of 0.25 mg/ml lysing enzymes (Sigma-Aldrich), 0.15 mg/ml chitinase (Sigma-Aldrich), and 0.01 mg/ml chitosanase (produced from Bacillus circulans) was added to the germinated spores and incubated with gentle shaking at 30°C for 2 h. Protoplasts were collected by centrifugation for 5 min at 200 × *g* at 4°C, washed twice with 0.5 M sorbitol, and resuspended in the same buffer. The incubation of protoplasts with the osmotic stabilizer sorbitol enables the regeneration of the cell wall, and during regeneration, cell wall constituents are released into the supernatant (45–47). The protoplasts were pelleted, and the supernatant was sterilized by filtration (0.22-μm filters) in the presence of protease inhibitors (Pierce). The supernatant was concentrated, and protein concentration was measured using the Bradford method (Bio-Rad). Negative-control samples were processed similarly, with the exception of the absence of protoplasts. Far-Western blot analysis using recombinant human GRP78 and anti-GRP78 antibodies was conducted to identify the *R. delemar* ligand.

### *In vivo* virulence studies.

For survival studies, equal numbers of male and female ICR mice (≥20 g) were purchased from Envigo and housed in groups of 5 each. Mice were immunosuppressed with cyclophosphamide (200 mg/kg intraperitoneally [i.p.]) and cortisone acetate (500 mg/kg subcutaneously [s.c.]) on days −2, +3, and +8, relative to infection. Mice were infected with 2.5 × 10^5^ in 25 μl *R. delemar* spores intratracheally. To confirm the inoculum, 3 mice were sacrificed immediately after inoculation, their lungs were homogenized in PBS and quantitatively cultured on PDA plates containing 0.1% triton, and colonies were counted after a 24-h incubation period at 37°C. Mice were treated with a single dose of 100 μg (i.p.) anti-β1 integrin antibody administered 24 h postinfection. Placebo mice received 100 μg of isotype-matched IgG. Mouse survival was monitored for 21 days, and any moribund mice were euthanized. Results were plotted using a log rank (Mantel-Cox) test.

### Study approval.

All procedures involving mice were approved by the IACUC of The Lundquist Institute for Biomedical Innovations at Harbor-UCLA Medical Center and in accordance with the NIH guidelines for animal housing and care. Human endothelial cell collection was approved by the institutional review board (IRB) of The Lundquist Institute for Biomedical Innovations at Harbor-UCLA Medical Center. Because umbilical cords are collected without donor identifiers, the IRB considers them medical waste not subject to informed consent.

### Statistical analysis.

Differences in GRP78 or integrin β1 expression and fungus-epithelial cell interactions were compared by the nonparametric Mann-Whitney test. In the survival study, the nonparametric log rank test was used to determine differences between isotype IgG control and the anti-integrin β1 Ab. Comparisons with *P* values of <0.05 were considered significant.
