# Supplementary figures and images for: GRP78 and Integrins Play Different Roles in Host Cell Invasion during Mucormycosis
Source: mBio. 2020 Jun 2;11(3):e01087-20. doi: 10.1128/mBio.01087-20 (PMC7267888; doi:10.1128/mBio.01087-20)

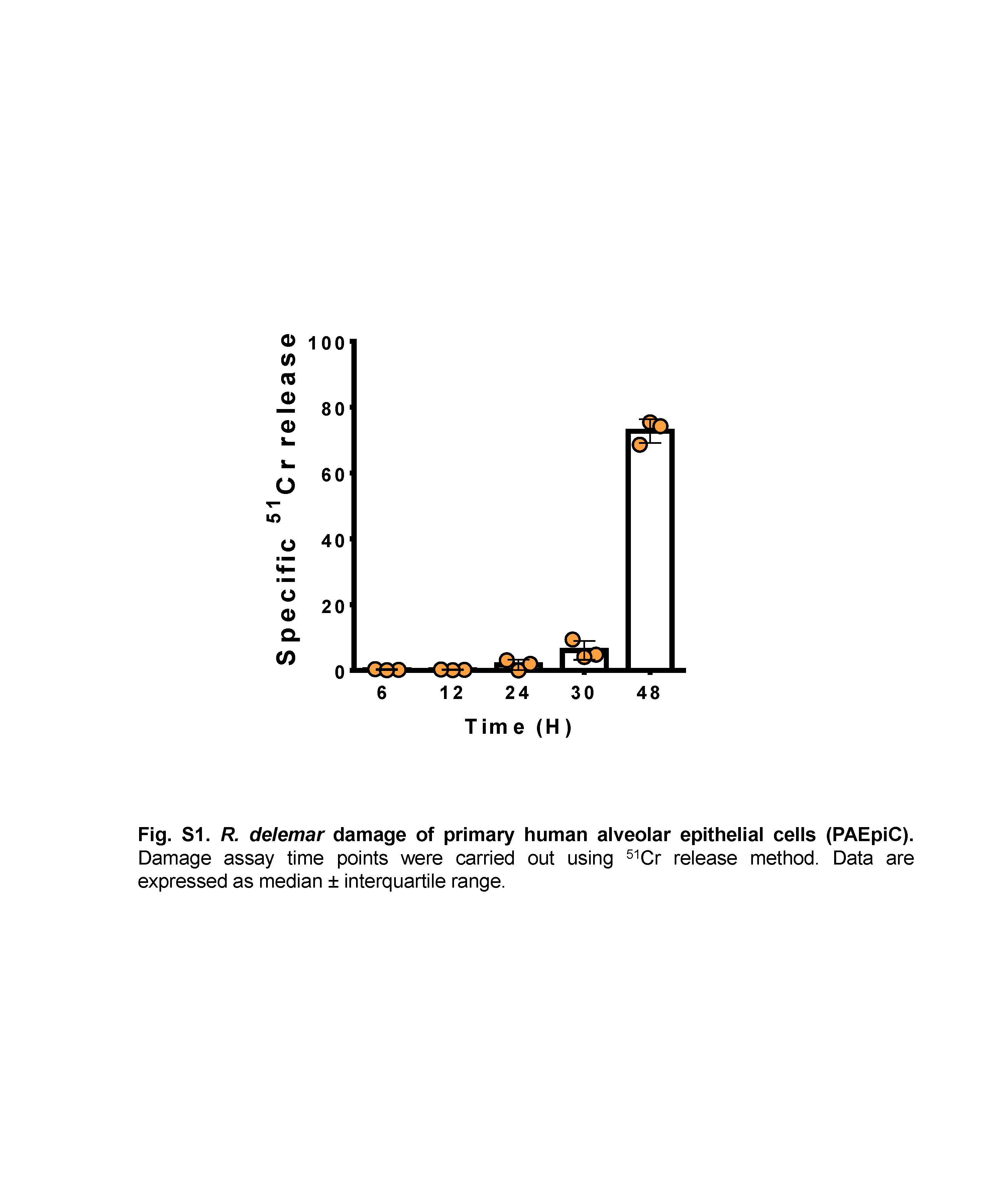

Supplement: FIG S1 [file mBio.01087-20-sf001.tif]

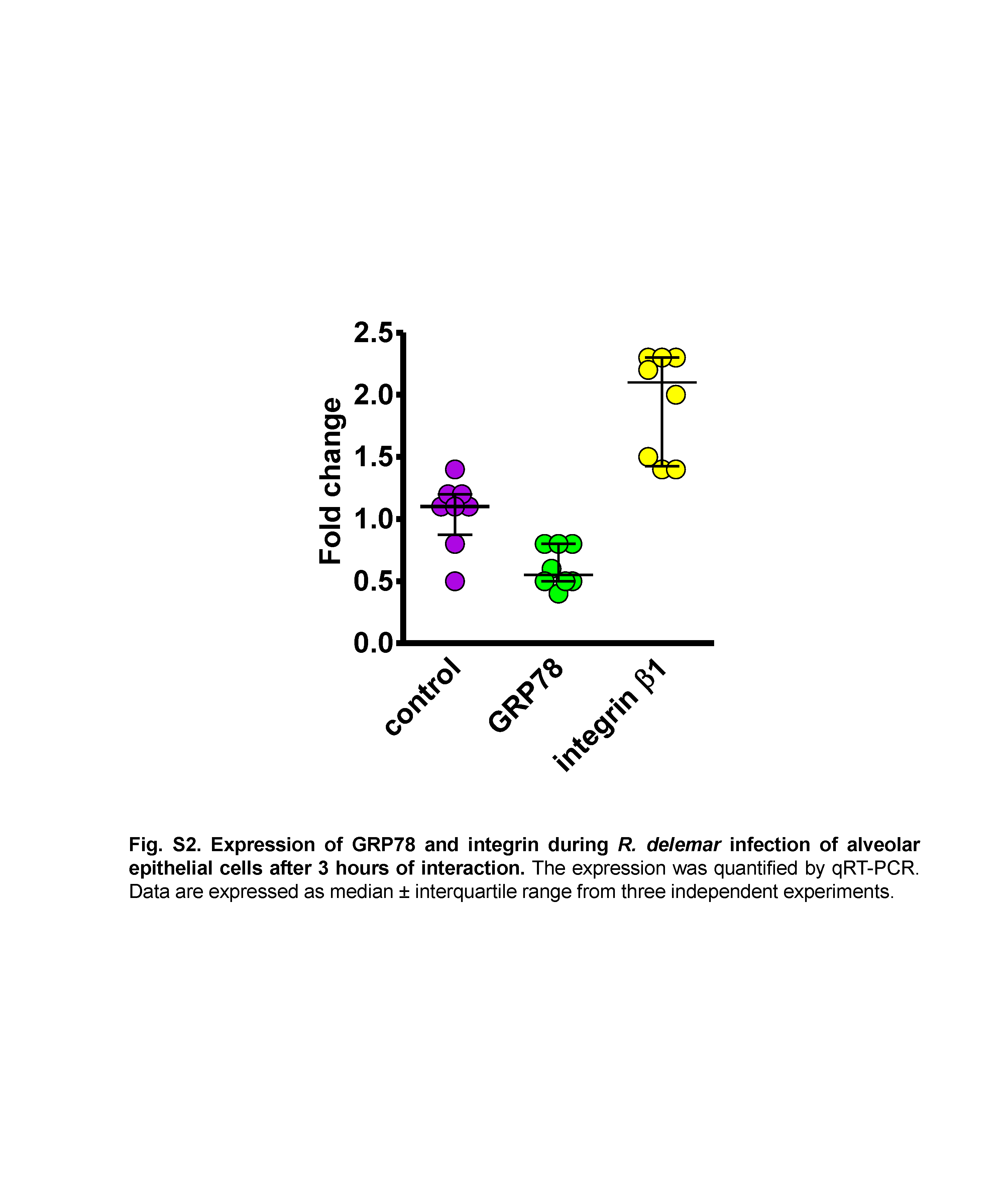

Supplement: FIG S2 [file mBio.01087-20-sf002.tif]

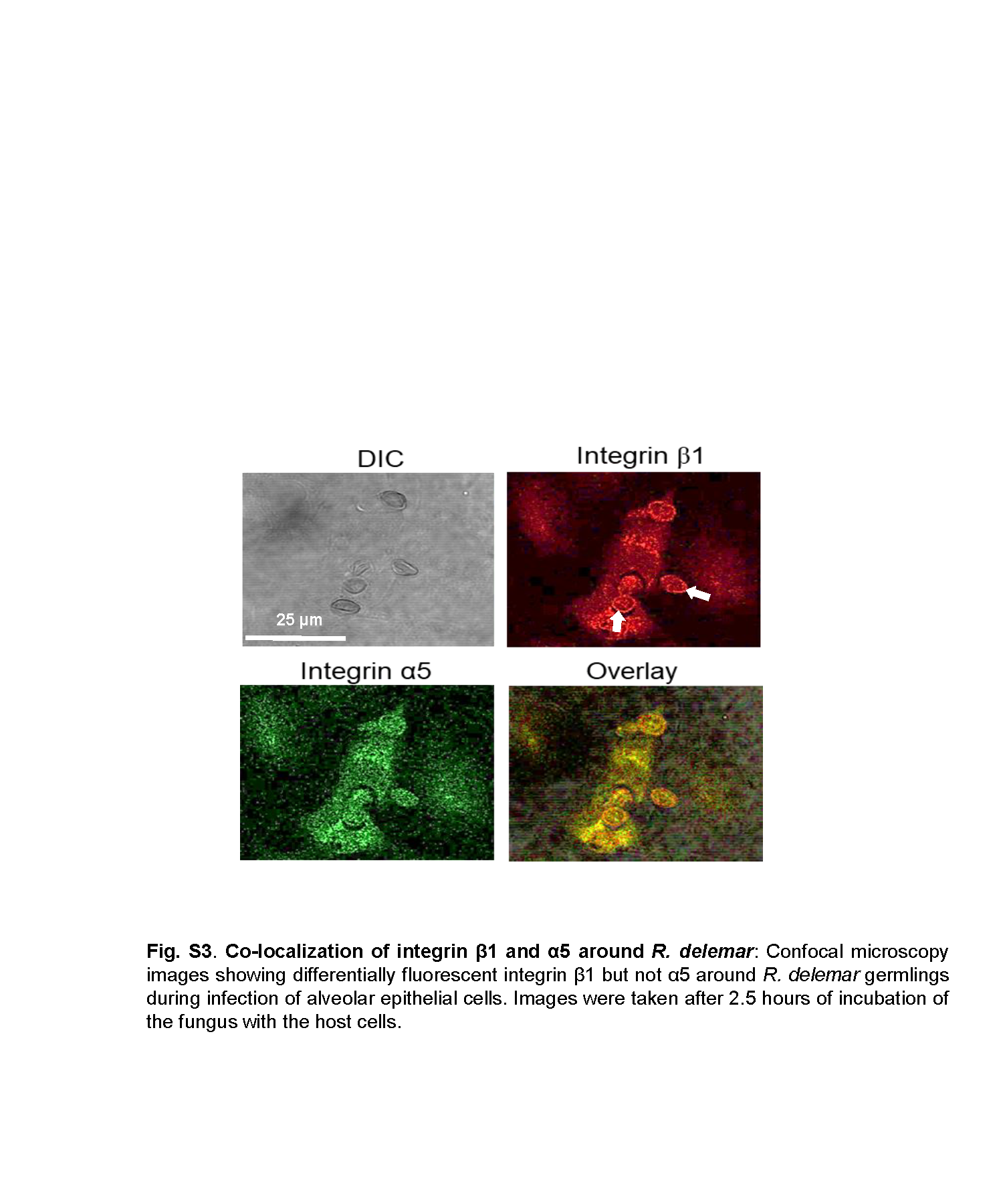

Supplement: FIG S3 [file mBio.01087-20-sf003.tif]

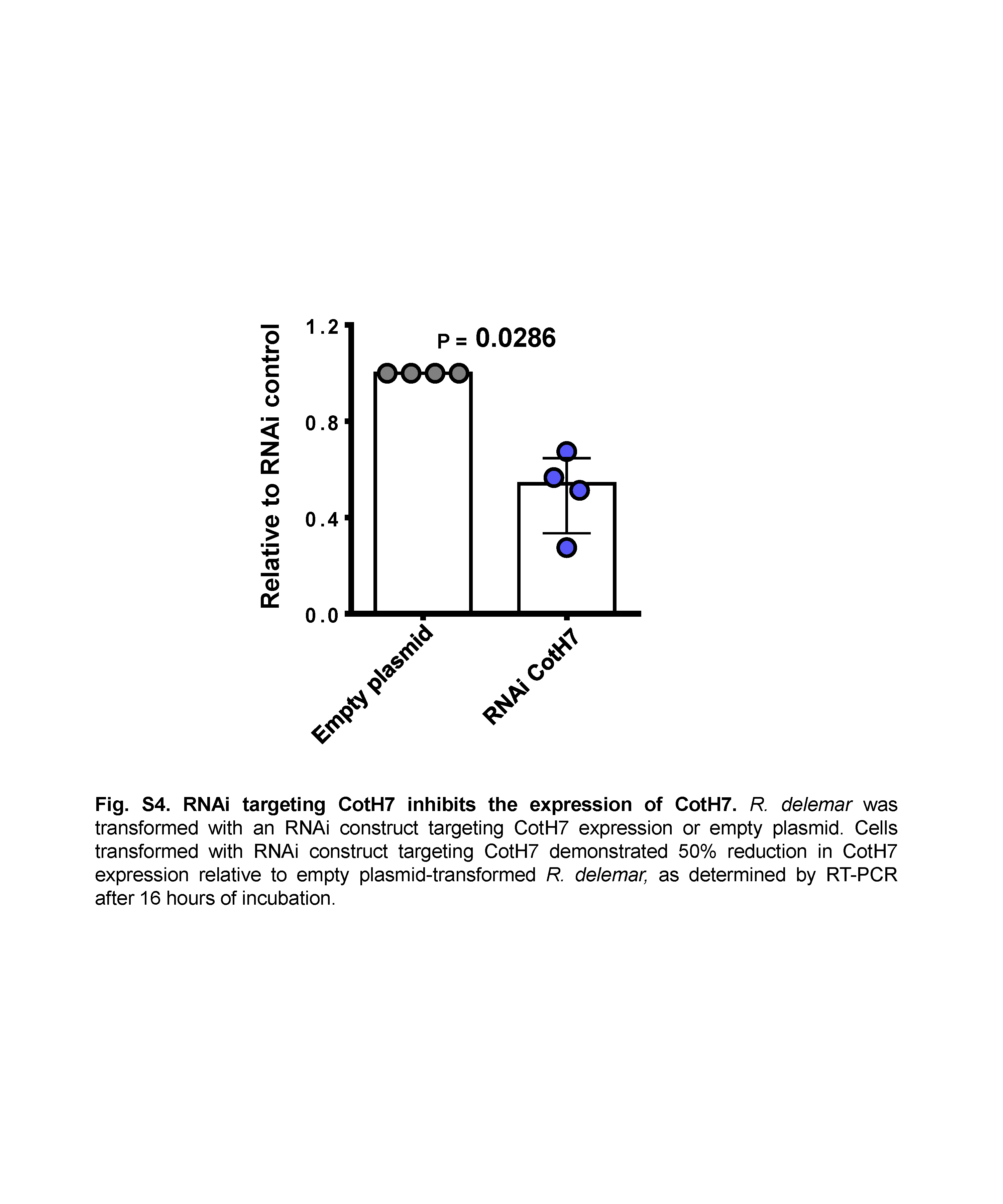

Supplement: FIG S4 [file mBio.01087-20-sf004.tif]

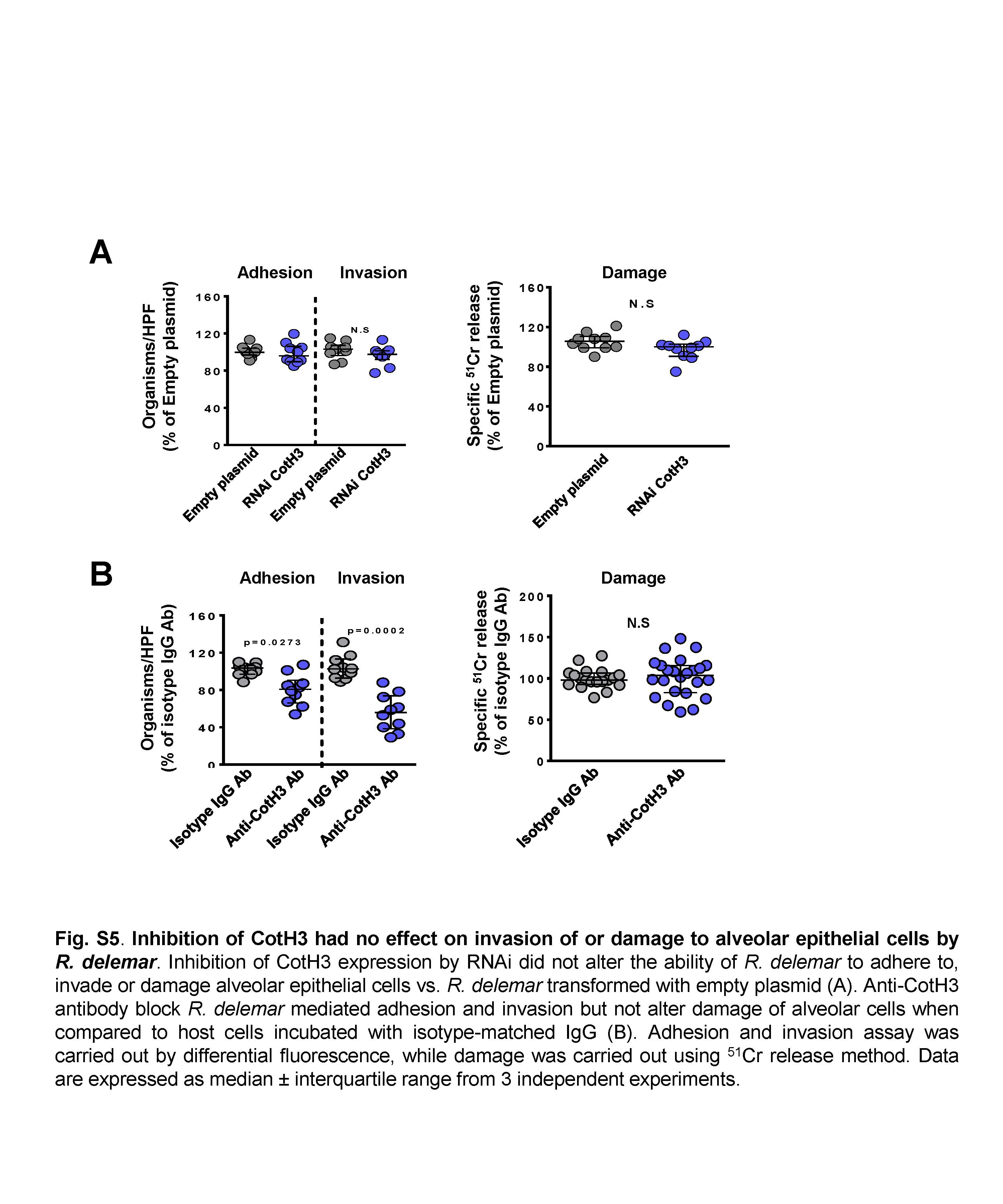

Supplement: FIG S5 [file mBio.01087-20-sf005.tif]

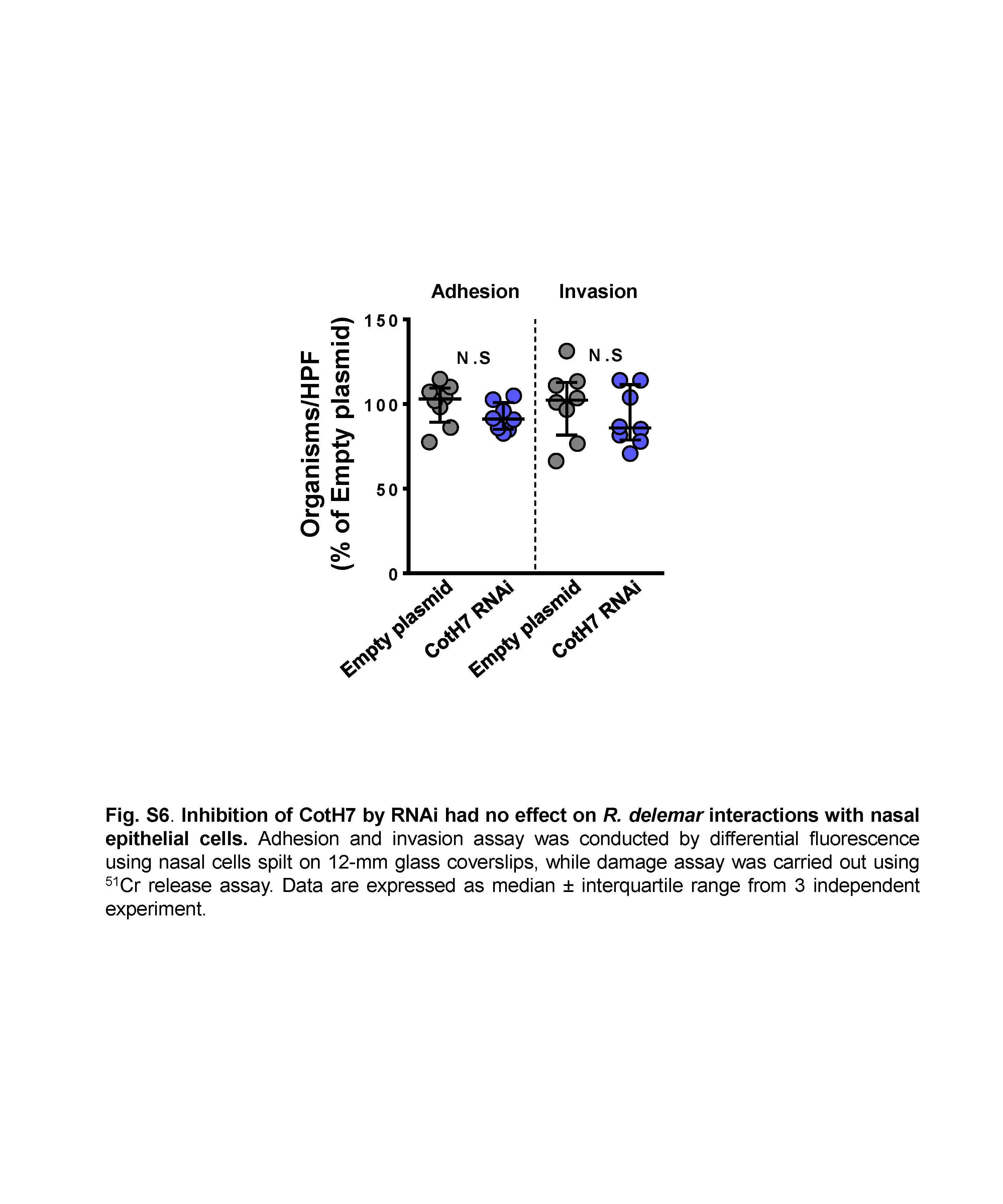

Supplement: FIG S6 [file mBio.01087-20-sf006.tif]

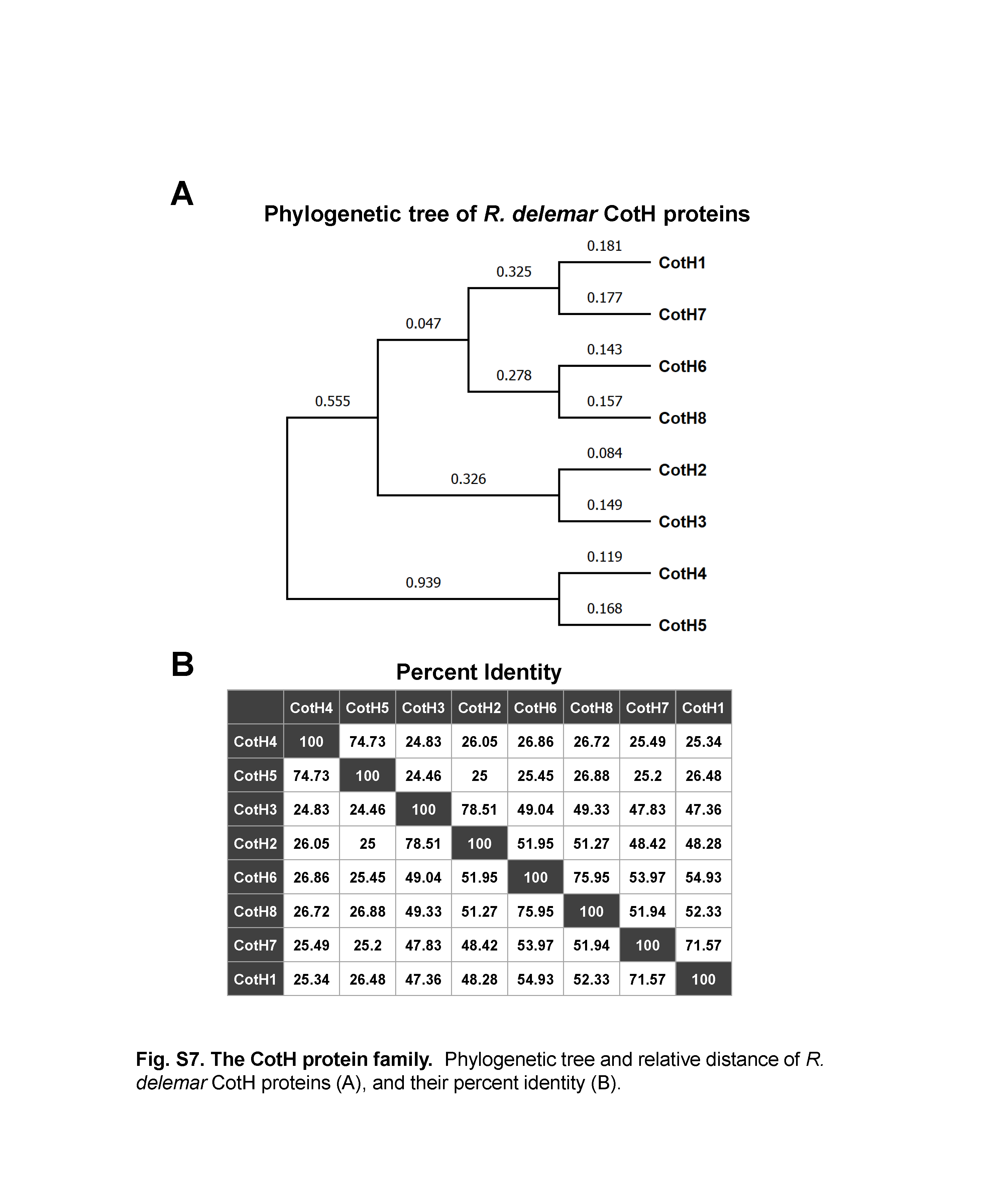

Supplement: FIG S7 [file mBio.01087-20-sf007.tif]

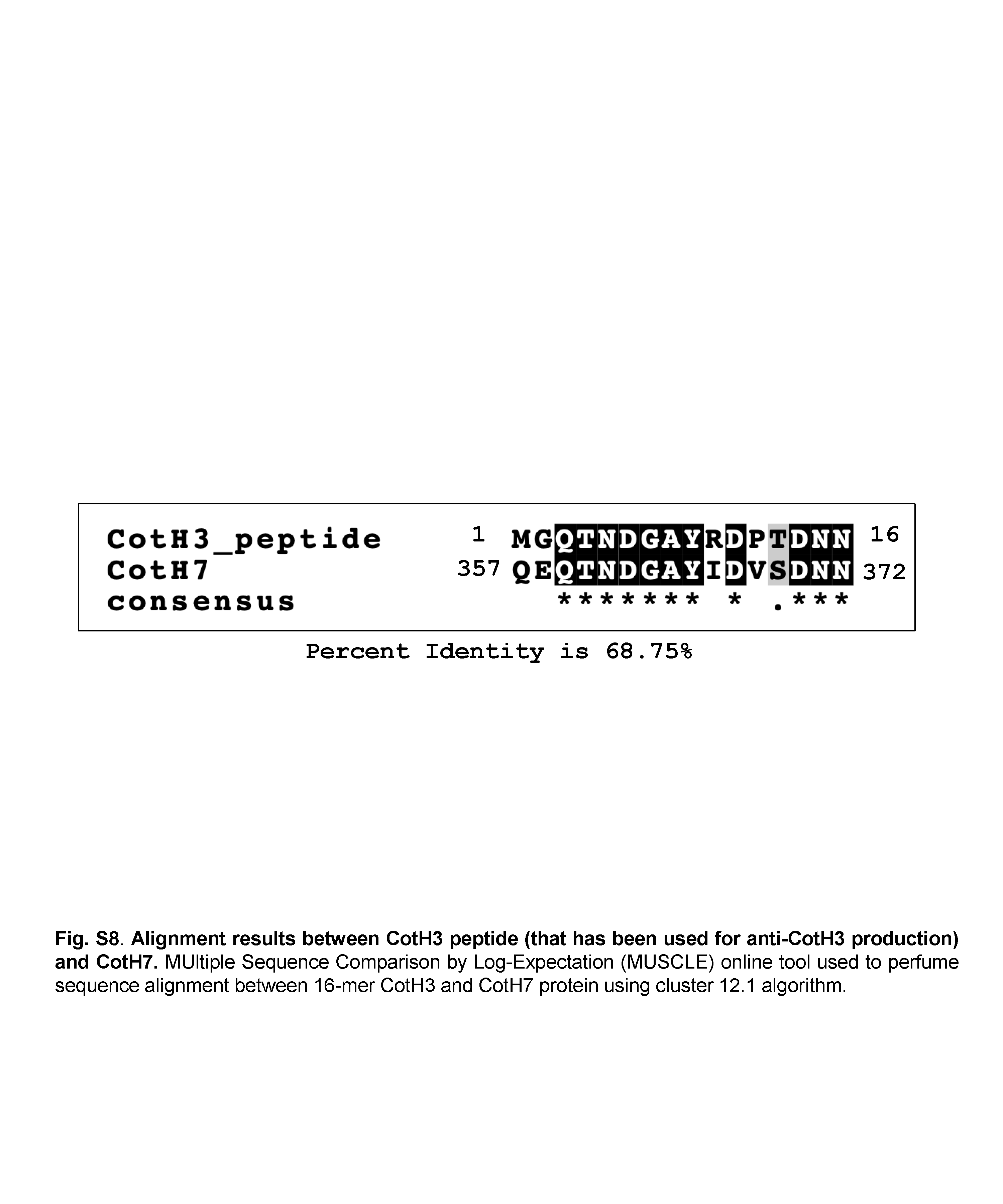

Supplement: FIG S8 [file mBio.01087-20-sf008.tif]
